# Cone Beam Computed Tomography in Oral and Maxillofacial Surgery: An Evidence-Based Review

**DOI:** 10.3390/dj7020052

**Published:** 2019-05-02

**Authors:** Robert Weiss, Andrew Read-Fuller

**Affiliations:** 1 Department of Oral and Maxillofacial Surgery, Texas A&M University College of Dentistry, Dallas, TX 75246, USA; 2 Baylor Scott and White Health, Baylor University Medical Center—Dallas, Dallas, TX 75246, USA; 3 Attending Physician, Baylor Scott and White Health, Baylor University Medical Center—Dallas, Dallas, TX 75246, USA

**Keywords:** cone beam computed tomography (CBCT), evidence-based, oral and maxillofacial surgery

## Abstract

Cone Beam Computed Tomography (CBCT) is a valuable imaging technique in oral and maxillofacial surgery (OMS) that can help direct a surgeon’s approach to a variety of conditions. A 3-dimensional analysis of head and neck anatomy allows practitioners to plan appropriately, operate with confidence, and assess results post-operatively. CBCT imaging has clear indications and limitations. CBCT offers the clinician 3-dimensional and multi-planar views for a more accurate diagnosis and treatment without the financial burden and radiation exposure of conventional computed tomography (CT) scans. Furthermore, CBCT overcomes certain limitations of 2-dimensional imaging, such as distortion, magnification, and superimposition. However, CBCT lacks the detailed depiction of soft tissue conditions for evaluation of pathologic conditions, head and neck infections, and temporomandibular joint (TMJ) disc evaluation. This review evaluates the evidence-based research supporting the application of CBCT in the various fields of oral and maxillofacial surgery, including dentoalveolar surgery, dental implants, TMJ, orthognathic surgery, trauma, and pathology, and will assess the value of CBCT in pre-operative assessment, surgical planning, and post-operative analysis when applicable. Additionally, the significant limitations of CBCT and potential areas for future research will be discussed.

## 1. Introduction

Many critical functions in oral and maxillofacial surgery (OMS) are served by 3-dimensional imaging. Clinicians frequently operate in areas of the face and jaws, which cannot be directly observed prior to a procedure, consequently risking damage to critical structures, such as nerves and blood vessels. Furthermore, complex surgical procedures, such as orthognathic surgery and treatment of traumatic injuries require meticulous preoperative planning. Therefore, it is important to utilize imaging modalities that provide detailed information that can ensure accurate diagnosis and good clinical outcomes.

Cone beam computed tomography (CBCT) has become a mainstay in oral and maxillofacial surgery, and many surgeons have a CBCT machine available in the office. CBCT offers the clinician 3-dimensional and multi-planar views for a more accurate diagnosis and treatment without the financial burden and radiation exposure of conventional computed tomography (CT) scans. Furthermore, CBCT overcomes certain limitations of 2-dimensional imaging, such as distortion, magnification, and superimposition.

While CBCT is used for many purposes, including routine OMS procedures, such as removal of impacted teeth and placement of dental implants, the quality of literature supporting its use and demonstrating superiority over other imaging methods is quite varied.

This review explores the evidence-based application of CBCT in the various fields of oral and maxillofacial surgery, including dentoalveolar surgery, dental implants, temporomandibular joint disorders, orthognathic surgery, maxillofacial trauma, and pathology. Each subsection will review the current literature in a clinical topic, including an analysis of the efficacy of CBCT in the pre-operative assessment, as well as the surgical and post-operative analysis, when applicable. In addition to providing an analysis comparing CBCT to other available imaging modalities, this review will discuss the perceived weaknesses of CBCT and explore areas that could benefit from further research.

## 2. Methods and Materials

A thorough critical review was conducted separately for each respective section encompassed within the field of Oral and Maxillofacial Surgery. Using PubMed as the primarily search database, emphasis was placed on high-evidence based articles, such as systematic reviews, meta-analysis, and large sample size randomized controlled trials. Unless very limited data existed, pilot studies, case series, and case reports were disregarded. While the keywords for each respective section varied slightly, those keywords that remained consistent were: CBCT, Cone Beam computed tomography, accuracy, comparison, and treatment outcome.

### 2.1. Dentoalveolar

In many routine dentoalveolar procedures, 2-dimensional plain films, including panoramic, periapical, and occlusal radiographs, are sufficient in the evaluation, treatment planning, and post-operative analysis. However, in specific situations, a more comprehensive 3-dimensional CBCT image is the radiograph of choice, specifically in assessing the position of impacted teeth and their position relative to vital structures including nerves and bony cortices [1,2,3]. Furthermore, complications, such as root displacement or fragmentation, are more accurately depicted with CBCT [4].

### 2.2. Impacted Third Molars

Surgery to remove impacted mandibular and maxillary molars, particularly third molars, may pose significant risks to nearby vital structures, including the inferior alveolar nerve (IAN), the maxillary sinus, vasculature, or adjacent soft and hard tissue.

### 2.3. Mandibular Third Molars

Panoramic radiography has long been considered the gold standard for initial evaluation of third molars in the context of adjacent vital structures [5]. In 1990, Rood evaluated seven radiographic signs commonly associated with an increased risk for IAN impingement or injury, which were later studied and further defined by other authors [6,7,8,9]. Radiographic signs include darkening, deflection, or narrowing of the root, dark/bifid apex, cortical interruption, diversion, or narrowing of the canal, only three of which (darkening of the roots, deflection of the canal, and interruption of the cortex) were found to be statistically significant [6].

Numerous articles have sought to answer a critical question when evaluating mandibular third molars: Is panoramic imaging alone sufficient to stratify the risk of inferior alveolar nerve injury? A large meta-analysis conducted by Sun et al. concluded that while panoramic radiography has high specificity, its sensitivity is inadequate for ruling out the risks of nerve damage [10,11,12]. While more recent articles illustrate the inadequacies of panoramic imaging in predicting IAN injury, they did not compare its sensitivity to CBCT or any other alternative method of imaging. While panoramic radiographs accurately detect troubling radiographic signs, the ability to rule out risks of IAN damage by panoramic imaging is fair at best [11,12].

CBCT provides coronal and sagittal dimensions of the relationship of the IAN and the mandibular third molar, which allows the clinician to appreciate the IAN proximity in a vertical, lateral, and depth dimension. This affords the clinician the ability to measure the exact distance in all dimensions, as well as evaluate a potential inter-radicular course of the IAN [1,13,14,15].

In a comparison between panoramic and CBCT imaging, Ghaeminia found no significant difference in the sensitivity and specificity of both modalities. However, CBCT allowed for the localization of the IAN in the bucco-lingual dimension. In instances where it may be located lingually, this may ultimately help to dictate surgical approach and technique [15]. In a systematic review, Matzen concluded that periapical (PA) or panoramic (PANO) examination is sufficient in most cases prior to removal. However, CBCT may be helpful when one or more signs for a close contact between the tooth and the canal are present on 2-dimensional imaging if it is believed that CBCT will change the treatment or the outcome of the patient. Only very few high-evidence studies on the efficacy of CBCT for radiographic examination of mandibular third molars exist, and in conclusion, periapical or PANO examination is sufficient in most cases before removal of mandibular third molars [3].

While several articles have addressed the use of CBCT imaging and its application to mandibular third molars, few evidence-based studies support the conclusion that CBCT alters clinical decision making, and other studies go as far as to conclude that CBCT does not decrease complications [3,16]. In most cases, panoramic or periapical radiographs are sufficient, but CBCT allows for better risk assessment of mandibular third molars in relation to the inferior alveolar canal, particularly in respect to the buccolingual dimension. In summary, the use of CBCT is at the clinician’s discretion when they feel it may provide additional useful diagnostic information about the IAN’s location, which may alter the surgical plan for removal. Currently, there remains limited definitive data to suggest the routine use of CBCT in the evaluation of mandibular third molars. Further high-level evidence-based studies may help to better define the prognostic application of CBCT [3,15].

### 2.4. Maxillary Third Molars

When evaluating impacted maxillary third molars, panoramic and periapical images are often inadequate in demonstrating their relationship to the maxillary sinus and adjacent tissues due to imaging distortion and superimposition [17,18,19,20].

CBCT has been shown to have several significant advantages over panoramic and periapical imaging in assessing the relationship of third molars to the maxillary sinus. When using CBCT, a root protruding into the sinus occurred more frequently in the buccal roots of the maxillary molars. The 2-dimensional view of panoramic and periapical imaging inevitably lacks this pertinent information in certain circumstances [18]. Hassan investigated the reliability of both periapical and panoramic radiographs in detection of tooth root protrusion in the maxillary sinus by correlating the results with cone beam computed tomography. In total, 638 teeth were examined, and clinicians were asked to classify the teeth based on their relationship to maxillary sinus wall. The results demonstrated that both PA and PANO radiographs are not reliable in determining the exact relationship between the apex of tooth roots and the maxillary sinus floor [21]. In another study, clinicians classified maxillary third molars in their relation to the sinus into four categories, thus comparing the efficacy of their subjective evaluation on PANO to CBCT [17]. Only 39% of the teeth roots that projected in the sinus cavity on panoramic radiographs showed protrusion into the sinus with CBCT, and panoramic radiographs showed 2.1 times the root projection, which was statistically significant. Bouquet found in a clinical study that 3-dimensional scans were more precise than the panoramic radiograph by 1.67 mm for measurement of the level of impaction, by 0.74 mm for bone height separating the third molar roots and the sinus, and by 2.26 mm for the length of roots in the sinus [20]. While these articles did not allude to any statistical relevance or oroantral communication, root proximity to the maxillary sinus is a known risk factor [17,18,21].

These studies provide sufficient data to support the advantages of CBCT when considering the extraction of upper third molars by providing both qualitative and quantitative information not available to 2-dimensional imaging, particularly when exposure of the maxillary sinus is of concern. While these articles support the increased accuracy in determining precise location and relationship to the maxillary sinus, they do not address the effects it has on treatment.

### 2.5. Impacted Canines

Impacted maxillary canines often present significant diagnostic and treatment challenges. When assessing an impacted canine, several characteristics must be accounted for and evaluated prior to selecting a desired treatment plan and surgical approach, including buccolingual location, tooth angulation in all dimensions, root dilaceration, and proximity or possible resorption of adjacent teeth, all of which are extremely variable factors [21,22].

Previously, panoramic imaging has been used in combination with occlusal and periapical films to provide multiple views. CBCT is more accurate than either horizontal or vertical 2-dimensional films in determining the exact location of ectopic maxillary canines [23]. Especially on the buccal surfaces of incisors, conventional radiography fails to depict root resorption [24]. There is a significant difference between 2D and 3D images regarding the width, angulation, and location of the canine, and amount of root resorption of adjacent teeth [25]. In a comparative analysis, Haney concluded that when CBCT was taken following the evaluation by PANO, treatment plans differed 27% of the time when planning surgical procedures on impacted maxillary canines [26].

CBCT imaging allows for a very accurate localization of impacted canines, providing important information regarding the inclination of the long axis of the tooth, bucco-palatal position, amount of bone overlying the tooth, resorption of adjacent teeth, condition of the adjacent teeth, and the stage of dental development [27,28].

### 2.6. Supernumerary Teeth

The presence of supernumerary teeth is a frequent odontogenic phenomenon, presenting clinicians with many options for treatment, including extraction, facilitated eruption, or retention. Similarly to impacted teeth, the 2-dimensional analysis does not provide information regarding the tooth’s true position, particularly in the bucco-lingual dimension, which can greatly influence the overall treatment plan. Liu et al. evaluated 626 supernumerary teeth, comparing the pre-operative analysis of several clinicians by 2-dimensional and 3-dimensional parameters, and found a statistically significant increase in accuracy in localization. CBCT imaging yields accurate 3-dimensional pictures of adjacent dental and bony structures, which is helpful for pretreatment evaluation of supernumerary teeth [29].

### 2.7. Conclusion

The advantages of CBCT in regard to evaluation and treatment pertaining to dentoalveolar surgery include increased visualization and exact location determination. Disadvantages of CBCT include cost and increased radiation exposure when compared to PANO.

From the presented research, it can be concluded that 3-dimensional imaging by way of CBCT can provide an increased visualization and enhance diagnostic capability but does not increase sensitivity and specificity when compared to plain PA and PANO radiographs when evaluating mandibular third molars [1,13,14,15] (Table 1). On the contrary, evidence exists demonstrating an increase in diagnostic capability of CBCT during the evaluation of maxillary third molars. The difference of CBCT’s role in maxillary versus mandibular third molars pertains to superimposition and distortion more commonly encountered in the posterior maxilla [17,18].

However, while clinically relevant for added diagnostic capability, the additional information provided by CBCT led to a change in the treatment plan only in respect to impacted canines. Clinical outcome and reduction in the number of complications, while addressed in few articles, were undetermined. Further research that explores the treatment outcomes and complication rates is indicated.

## 3. Dental Implants

Proper selection of preoperative imaging is a critical step in the evaluation and treatment planning process prior to implant surgery. In virtually all regions of the jaws, there are critical anatomic structures that require careful evaluation in three dimensions. The American Academy of Oral and Maxillofacial Radiology (AAOMR) currently advocates for the use of cross-sectional imaging for all dental implant surgeries. [30] Although surgeons still rely on 2-dimensional imaging in routine cases, the literature does illustrate that CBCT offers significant advantages that can alter treatment planning and improve surgical outcomes.

### 3.1. Assessment and Planning

#### 3.1.1. Anatomy

Preoperative analysis and surgical planning of implant placement demands close attention to a number of variables, and numerous anatomic considerations must be taken into account based on the anatomic location.

In the anterior maxilla, the nasal floor, buccal concavities, and nasopalatine canal present anatomic obstacles for precise implant placement. In a comparative analysis, Bokkasam concluded that panoramic radiography is reliable in assessment of the nasal floor and maxillary sinus, provided that the position of the patient, image distortion, and the inherent magnification factor are taken into consideration. However, he also found CBCT had a significant advantage when measuring alveolar bone height in the nasal floor region due to superimposition of the hard palate [31]. When immediate implant placement is planned following tooth extraction in the anterior maxilla, CBCT is preferred due to the capability of measuring buccal alveolar bone width [32] (Table 2).

In the maxillary premolar and molar region, the quality of bone and proximity of the maxillary sinus are key considerations. The thickness of buccal and lingual cortices, vertical height in relation to the maxillary sinus, and incidence angle of the alveolar bone in respect to the occlusal plane can all be best visualized with CBCT [33]. While panoramic imaging may approximate vertical height in select locations, all other aforementioned findings cannot be assessed adequately with 2-dimensional imaging alone. As referenced previously in the dentoalveolar section, Sharan demonstrated the efficacy and superiority of CBCT in regard to the intricacies of root proximity to the maxillary sinus [17]. When more precise information is needed regarding anatomic considerations of the posterior maxilla in respect to implant placement, CBCT is the imaging modality of choice [17].

In the mandible, while the recipient bone is often of greater density with thicker cortical plates, significant vital structures, including the IAN canal, submandibular gland concavity, mental foramen, and variations in bony and neurovascular architecture, must be considered and evaluated in each patient. CBCT-reformatted panoramic images have been shown to be superior to orthopantomogram in identifying the mandibular canal, because these images are free of magnification and superimposition [34]. In a retrospective study, Uchida examined the maximal mesial distance of the anterior loop of the IAN in order to determine a safe distance for implant placement in the anterior mandible. Due to the large variation in the course and diameter of the IAN, a minimum distance could not be recommended, further supporting the use of CBCT to evaluate patients’ anatomy on a case-by-case basis [35].

In the posterior mandible, the submandibular gland concavity poses a risk for perforation through the lingual cortex during implant osteotomy leading to injury of adjacent vital structures. The majority of mandibles possess a lingual concavity in the posterior aspect, whereas in the anterior mandible, buccal and lingual cortices are parallel [36]. The mean undercut depth of the lingual concavity and angulation at a level 2 mm above the IAN canal is, on average, 2.4 mm and 57.7°, respectively [37]. The anatomic location and the degree of the lingual concavity adds vital information that can influence implant treatment planning in the posterior mandibular region. A retrospective analysis of over 400 mandibles demonstrated a statistically significant difference in apparent vertical height of available bone when CBCT was compared to panoramic imaging. Therefore, alveolar bone height may be unpredictably inadequate in the posterior mandible based on panoramic imaging alone, and some authors, therefore, recommend CBCT for all prospective implant sites [38].

#### 3.1.2. Bone Quality and Volume

The volume and quality of bone are both essential to implant success. While many articles reference the importance of bone quality in implant surgery, current literature lacks a universally accepted system for grading bone quality based on objective measures [39]. The development of such a grading scale for bone quality may ultimately prove to aid in the preoperative implant planning process and help predict overall success rates.

Bone volume, on the other hand, is well defined and accurately calculated with the application of 3-dimensional imaging, such as CBCT [40,41,42]. Imaging software allows clinicians to characterize the orientation of the alveolar ridge, precisely measure the width at various locations, and identify anatomic or pathologic limitations. CBCT additionally provides value in evaluating alveolar fenestrations and dehiscences, both of which may alter treatment [40].

In areas of diminished bone density, such as the posterior maxilla, bone volume is even more essential in presurgical assessment and planning. In a study comparing volumetric measurements of CBCT images to anatomic specimens, Bou found CBCT to be very accurate in predicting bone volume in the posterior maxilla [39]. Numerous other articles confirm that CBCT provides precise measurements in evaluating bone height and volume in the maxilla and mandible, to within 0.6 mm in each dimension [40,41,42,43], (Table 2).

#### 3.1.3. Implant Selection

Selection of the appropriate implant is based on several criteria, including operator preference, planned restoration, anatomic location, and a variety of other subjective measures. Articles portraying selection bias or subjectivity in implant selection based on personal preference were excluded from this review.

Determining implant width is done primarily by clinical evaluation and measurement of the surgical site [44]. However, depending on the location, length of the implant selected by the surgeon may vary depending on the imaging modality used.

Schropp’s work demonstrated that implant selection differs depending on the image modality used, finding that surgeons alter their selected implant size 70% of the time when 3-dimensional imaging is provided in addition to a traditional orthopantomogram. Furthermore, the surgeons in his study used the predetermined implant size in only 33% of cases planned with panoramic imaging, compared to 87% planned with CBCT, demonstrating CBCT’s superiority in clinical accuracy [45]. Correa compared implant selection between CBCT, panoramic images reconstructed from CBCT, and conventional orthopantomograms. In the premolar regions, there was no significant difference. However, a significant difference in implant length and width was observed more posteriorly in the mandible, with CBCT planning resulting in the selection of a smaller width and length [46].

In a study of over 1500 patients, Vazquez determined that while differences may exist when using the different imaging modalities, panoramic radiography appears to be sufficient in determining implant length when a safety margin of at least 2 mm above the mandibular canal is respected by the surgeon [47] (Table 2).

#### 3.1.4. Augmentation and Site-Preparation Procedures

Many clinical scenarios warrant additional procedures to augment the bone surrounding an implant site, either at the time of implant placement or during a separate surgery including, possibly during extraction of the tooth planned for replacement. While not comprehensive, commonly-used augmentation procedures include alveolar bone grafting for site preservation, maxillary sinus lifts, and ridge splitting. Alternatively, zygomatic implants may obviate the need for augmentation surgery when posterior maxillary restorations are planned and there is limited bone available. Regardless of the proposed method of addressing a site deficient in bone, CBCT is the indicated imaging modality in the preoperative assessment process [30].

Guerrero evaluated 365 implants planned with either CBCT or panoramic imaging and found that CBCT offered more spatial information to the clinician, leading to more accurate prediction of the need for alveolar bone grafting at the time of implant placement [48]. Another study sought to evaluate linear alveolar bone levels and extraction socket circumferential dimensions and found a statistical difference depending on the radiographic method used. The authors concluded that circumferential measurements of extraction sockets performed on panoramic imaging were inaccurate compared to periapical or CBCT images [49].

Placing implants in the posterior maxilla demands close attention to the maxillary sinus and its anatomic relation to available bone height and volume. Baciut examined the pre and post-operative assessment of sinus grafting procedures in the context of implant placement with either CBCT or panoramic imaging. Minimal difference was observed in post-operative evaluation, however, there was a significant increase in surgical confidence and prediction of complications when using CBCT, as PANOs frequently overestimate bone quality and quantity [50]. While panoramic radiographs may show excessive variability or an overestimation bone height, they can adequately determine the need for vertical augmentation procedures. However, in specific cases in which lateral alveolar bone is deficient, CBCT may be more appropriate [51]. Septation in the maxillary sinus, which can only be reliably found using 3-dimensional imaging, is present 47% of the time, further justifying the routine use of CBCT prior to sinus augmentation procedures [52]. The location of the arterial supply to the sinus is also an important anatomic consideration. There is a visible canal on CBCT imaging 32.9% of the time in the palatal canine region and 49.5% of the time in the lateral maxillary sinus wall, and the use of CBCT can avoid injury to these structures [53].

When zygomatic implants are used in place of alveolar dental implants due to limited available bone, CBCT can be utilized to fabricate drilling guides to facilitate accurate implant placement due to the length of the implants and the anatomic intricacies of the zygoma [54]. Aparicio proposed a classification system used in treatment planning for zygomatic implants, in which CBCT is necessary in order to portray the 3-dimensional anatomy and relationship to adjacent structures [55].

Overall, in the preoperative assessment of dental implant surgery, there is significant evidence to support the use of CBCT in defining pertinent anatomy, alveolar bone volume, and precise implant size selection. Panoramic imaging is an unreliable radiographic technique unless meticulous precautions are taken for reproducible positioning of the patient. However, while CBCT undeniably provides additional information not well visualized in 2-dimensional imaging, PANO may be sufficient in select cases. In the few articles that examined treatment outcomes, 3D imaging information did not significantly alter the surgical plan that was based on 2D panoramic radiography. Ultimately, the use of CBCT remains up to the discretion of the provider, as no definitive evidence exists that demonstrates a true alteration in treatment outcome [30,45,56].

### 3.2. Application of Surgical Guides and Splints

With increased application of CBCT in dental implant planning, many clinicians have utilized this technology to fabricate custom guides to be used during surgery. CBCT can aid in eliminating possible manual placement errors and better match planning to prosthetic requirements [56]. Ganz discussed the numerous advantages that CBCT offers in surgical planning of implant placement, as previously mentioned. He strongly argues the use of guides in implant placement, concluding that the surgical guide ultimately offers a “link between the plan and the execution of that plan” [57].

However, the use of surgical guides continues to be a subject of debate, and additional data is needed to establish conclusive guidelines and indications [58]. A surgical guide poses many challenges that must be addressed prior to its universal application in implant surgery, mainly pertaining to material thickness and operator access. Specifically in the posterior jaws, limited opening and thickness of the guide may limit access and render placement difficult, if not impossible [59].

There is contradictory evidence pertaining to the accuracy of 3-D guides and method of fabrication. While guides can be constructed with stone models and laboratory materials alone, these merely direct the osteotomy at the crestal location of the desired implant. Therefore, they can provide a false sense of security to the surgeon, as they rely on the free-hand precision of the clinician for proper mesiodistal or buccolingual angulation. In an analysis comparing guides fabricated from dental models versus CBCT data, Boyoung found a smaller deviation in final implant location when using guides constructed from models [60]. Conversely, Besimo found no difference in crestal implant location deviation, and advocated for the use of CBCT-constructed guides due to the additional clinical information they provide, including more accurate angulation [61].

In a systematic review and meta-analysis, Yolanda evaluated the accuracy of surgical guides when the support relied upon tissue, tooth, and bone. Implant angle deviation, crestal position, and apex location were all recorded. The authors concluded that mucosa and tooth supported guides demonstrated a comparable and superior accuracy in relation to bone supported guides [62]. In another meta-analysis, Schneider found no significant difference regarding the method of template production or type of support [63].

Other studies have demonstrated that implant guides become more accurate if they are fabricated with CBCT planning data overlaid on dental models or merged with intraoral scans [64], and the accuracy of implant placement using this method is significantly greater than free-hand insertion [59,65].

### 3.3. Post-Operative Evaluation

Routine evaluation of implant success following placement is dependent on a number of factors. Aside from clinic examination, radiographs can help to determine the surrounding alveolar bone support and monitor progression over time. However, intraoral radiographs are subject to patient positioning, superimposition, and angulation changes, and consistency in technique becomes even more pivotal to serial evaluation and implant maintenance.

Corpas compared intraoral radiographs and CBCT images to histological analysis in order to detect bony changes following implant placement and found that minute bony changes in the short-term postoperative period can be estimated accurately with intraoral radiographs. Tissue parameters correlated significantly between histological and intraoral techniques, while CBCT could not provide this level of definition [66]. CBCT detects peri-implant circumferential, intra-bony, or fenestration defects, but is deficient in depicting dehiscences [67]. In a meta-analysis reviewing articles from 1991 to 2016, Bohner evaluated the use of CBCT, intraoral radiography, computed tomography, and panoramic radiography in regard to dental implants and bony evaluation. Taking into account positive predictive value, negative predictive value, diagnostic odds, and likelihood ratios, it was concluded that both CBCT and intraoral radiography demonstrated clinically acceptable assessment of peri-implant bone levels [68].

In summary, certain scenarios warrant the use of CBCT. If tooth removal is anticipated and augmentation procedures are required, CBCT is recommended. Additionally, if symptoms of pain or paresthesia persist, further evaluation may require CBCT analysis [30]. CBCT offers buccolingual visualization, volumetric bone data, and morphological assessment of implants. As Jacobs summarized in a systematic review, there is currently no definitive evidence to support the standard use of CBCT for postoperative evaluation of peri-implant bone. At this time, with certain exceptions, intraoral radiography should remain the main diagnostic imaging modality in monitoring implants post-operatively [69].

### 3.4. Conclusion

Due to the limited margin for error in the placement of dental implants, CBCT presumably serves an important role. Regarding diagnostic planning and analysis of local anatomy, bone volume, and implant selection, CBCT has been proven to be superior and more reliable than PANO (Table 2). Such examples, as mentioned previously, include measuring buccal and lingual cortices, visualizing and analyzing the lingual concavity in the posterior mandible, and defining a more accurate distance to the inferior alveolar nerve [31,32,41,42,43,49]. In regard to treatment, there were only a few categories in which CBCT significantly altered a predetermined plan: implant size selection and the necessity for bone augmentation at the time of implant placement. Limited data has quantified the ability of CBCT to affect clinical outcome or complication rates.

## 4. Pathology

In the broad scope of pathology encompassed within the head and neck region, there are certain lesions that can be assessed effectively by CBCT. Aside from the obvious benefit of analysis in three dimensions, CBCT offers an opportunity for a clinician to track growth change, appreciate borders in a depth perception that may otherwise be difficult to discern, and analyze relative approximation of adjacent vital structures [70,71,72].

The evaluation of malignant lesions is usually inadequate with CBCT alone and is limited mainly to the osseous extent of such lesions. MRI or medical grade CT (including intravenous contrast) with a soft tissue window are the imaging modalities of choice when assessing a lesion originating from, or involving, the soft tissue of the head, neck, or oral cavity [70]. However, while CBCT is limited in soft tissue analysis, as previously described, it does possess certain clinical applications in the evaluation of malignancies [73,74,75]. Conversely, benign lesions of soft tissue demonstrate poor diagnostic potential with CBCT [30,76]. As such, focus of this review will pertain to the osseous components of lesions, including cysts, tumors, and cancers with bony involvement.

### 4.1. Pre-Operative Analysis

Pathologic lesions in the jaw can be found incidentally on panoramic imaging. Once the presence of a lesion is determined, however, it is up to the clinician to decide if additional imaging is necessary. The 3-dimensional benefits of CBCT are well established in the literature, based on its relative accuracy within hard tissue [42,77,78]. This proves beneficial in specific situations in which adjacent anatomy and size of the lesion become increasingly important [79].

In a comparison between CBCT and panoramic imaging, Cheunchompoonut compartmentalized jaw lesions into 4 categories based on definition of the border of the lesion. Panoramic imaging was determined to be reliable when the borders of the lesion are well defined, respective of size and location [77]. In comparison to panoramic imaging, CBCT offers the ability of DICOM file transfer for 3D printing as an aid in surgical planning and treatment [71]. However, no current literature validates improvement in surgical outcome when a 3-dimensional modeling is used.

Lim conducted a comparative analysis of 12 different types of commonly encountered osseous cysts or tumors in order to determine diagnostic potential between PANO and CBCT. While CBCT provides the benefit of defining intraosseous lesions in all three dimensions, it does not improve clinical outcome [76,80,81].

Detecting the bony extension of oral malignancies is of utmost importance for staging of the lesion and for surgical planning. Dreiseidler concluded in a prospective study that CBCT is comparable to multi-slice CT and single photon emission in predicting the bony involvement of malignancies encompassed in the oral cavity [72,74]. Furthermore, CBCT is proven to be superior in the osseous invasion of intraoral squamous cell carcinoma when compared to panoramic imaging [75].

### 4.2. Progression and Post-Operative Follow Up

In certain circumstances, a non-surgical approach to a pathological lesion involving close monitoring may be appropriate. Additionally, recurrence rates of certain benign osseous lesions are rather high, and routine postoperative re-imaging is paramount for early detection of recurrences or expansion of known lesions. Although CBCT has numerous advantages in the initial diagnosis of intraosseous lesions, the added benefit of the third dimensional analysis is dependent upon the nature of the planned treatment. When screening for recurrence after complete enucleation or resection of an osseous lesion, PANO is thought to be sufficient [82].

### 4.3. Conclusion

Advantages of CBCT when evaluating pathologic lesions of the head and neck region include providing a high-quality image with minimal distortion as a decreased cost and lower radiation exposure when compared to MRI and CT. Disadvantages include a very apparent lack of soft tissue definition, thus limiting its overall applicability as a treatment modality of choice for pathology.

In conclusion, PANO and CBCT both provide sufficient imaging for most odontogenic osseous lesions [71,72]. CT or MRI is indicated if there is evidence of extraosseous soft tissue involvement or suspicion of malignant potential [70]. In reality, differential diagnosis of pathologic lesions is rarely limited to radiographic interpretation alone, and important information can be obtained in the history and physical examination of the patient.

Advantages of CBCT are that it offers the clinician the ability to make precise measurements of an osseous lesion [71,72]. It also affords the opportunity for continued monitoring, tracking the progression or recurrence of a lesions removed. Similarly to CT and MRI, CBCT offers a 3-dimensional analysis of adequate quality at a reduced radiation exposure. However, current literature provides insufficient evidence to indicate the advantage of CBCT to other 3-dimensional imaging modalities in regard to diagnosis, treatment, outcome, or disease monitoring and maintenance, but the surgeon should consider the overall benefits of CBCT, including decreased cost and radiation exposure, when MRI or CT are not clearly indicated.

The variability of all the possibly pathologic lesions of the head and neck makes quantifying the role of CBCT in diagnosis, treatment, and outcome extremely difficult. Many of the articles reviewed compare CBCT to other 3-dimensional imaging modalities and found no significant difference in diagnosis or definition of the lesion.

## 5. Inflammation and Infection

### 5.1. Hard Tissue

While acute inflammatory changes are difficult to depict on any radiographic study, hallmark signs of chronic inflammation of the jaws include periosteal layering and disruption of the bony cortices. CBCT allows for increased accuracy in visualizing periosteal reactions, compared to previously used periapical, occlusal, and panoramic plain film radiographs [83].

### 5.2. Osteomyelitis and Osteonecrosis

Necrosis of the jaw, regardless of etiology, has characteristic radiographic findings that include osteosclerosis, osteolysis, dense woven bone, thickened lamina dura, subperiosteal bone deposition, failure of postsurgical remodeling, mottling, sequestra formation, and subsequent pathologic fracture if progressive and left untreated [83,84,85,86]. Clinical features include exposed non-vital bone or draining fistulas. As demonstrated in numerous studies, CBCT imaging is more accurate in depicting radiographic changes than panoramic radiographs [85,87]. Olutayo retrospectively compared radiographic methods for detecting osteonecrosis at various stages or degrees of severity at initial presentation. CBCT was able to fully characterize the extent of bony lesions in cases where necrosis was very minimal, whereas panoramic imaging was deficient. The authors concluded that CBCT can better identify and characterize osteonecrosis and could prove more beneficial in disease management [85]. Similarly, Treister compared clinical and radiographic features of patients with bisphosphonate-associated osteonecrosis of the jaw (BONJ). CBCT is superior to PANO in its ability to characterize the nature and extent of radiographic changes in BONJ or suppurative osteomyelitis [86,88]. However, clinical significance remained unclear, and they were, therefore, unable to recommend specific radiographic guidelines [86].

Schulze compared cases of osteomyelitis diagnosed CBCT with histological confirmation, and while the sample size was small, CBCT was found to provide a positive correlation and accurate diagnosis of osteomyelitis [89].

Accepted guidelines for selecting radiographic studies in the setting of suspected bony infection and inflammation do not exist currently. However, as Arce suggests in his expert review article, imaging is an essential part of the clinical assessment of osteonecrosis and might be an additional tool for tracking the progression of the disease [84].

### 5.3. Soft Tissue

Rapid, accurate diagnosis of fascial space infections of the head and neck is paramount to guide surgical management. Adequate imaging should define boundaries of the infected region, discern between abscess and cellulitis, and monitor disease progression [90,91]. The application of CT helped to pioneer the understanding the pathways of the spread of deep facial infections [90,91]. Due to its wide field of view and accuracy in soft tissue detection, CT with intravenous contrast is regarded as the most reliable technique for assessing fascial space lesions or abscesses. The addition of contrast agents allows for image enhancement of fluid collections, which is a feature not often available with CBCT [90].

Fourie et al. found in an anatomic study that CBCT adequately measured soft tissue thickness in the facial region but recommended a 0.3 mm voxel size for enhanced accuracy. [92] However, the benefit of contrast media for image enhancement makes CT the imaging modality of choice when evaluating soft tissue infections of the head and neck [90], and current literature does not advocate the use of CBCT for accurate detection and characterization of soft tissue infections.

### 5.4. Conclusion

Similarly to the pathology section, the application of CBCT in the diagnosis of infection or inflammatory changes in the field of Oral and Maxillofacial Surgery drastically varies dependent upon the tissue of examination.

CBCT has proven to be superior to PANO in the diagnosis of hard tissue inflammatory change or infection, such as osteomyelitis, offering a quicker diagnosis and more accurately staging the disease [90,91]. However, in the context of soft tissue, CT and the added benefit of contrast media remains far better in visualization and diagnosis than CBCT [90]. Changes in treatment plan and complications were not explored in any of the studies and clinical outcome could not be determined based on the current literature.

## 6. Temporomandibular Joint

Magnetic Resonance Imaging (MRI) remains the gold standard for comprehensive intra-articular evaluation of the temporomandibular joint, including disc anatomy, position, and movement, as well as the joint capsule, surrounding soft tissues, and musculature [93]. However, while MRI provides invaluable information, it remains inferior to computed tomography in providing detailed analysis of bony architecture [93]. Using osteoarthritis as the study focus, Ahmad compared PANO, CT, and MRI imaging. Positive percent agreement was recorded to be only 19% for PANO, while MRI and CT found a positive percent agreement of 59% and 84%, respectively. Thus, it was concluded that tomography was most predictive of condylar bony changes, while MRI was reserved for disc position and joint effusion analysis [94]. Although this study did not focus on CBCT specifically, the comparable advantages to CT, and the greater availability of CBCT in the OMS clinic setting may make it useful in the diagnosis of osteoarthritis of the jaw.

CBCT has been shown to improve overall diagnostic accuracy for internal derangement when used as a supplement to MRI [95]. Mohammed compared the interpretation of TMJ disorders when evaluated by MRI alone versus CBCT in addition to MRI and found that inter-examiner and intra-examiner consistency was higher when CBCT was used in addition to MRI when compared to MRI alone.

### 6.1. Imaging Methods

Panoramic radiographs have been regarded as having a low reliability for condylar change and low accuracy in assessment of the temporal bone and glenoid fossa when compared to CBCT [96,97]. Comparing PANO and CBCT, Dahlstrom concluded that there is a marked variability in analysis amongst observers when viewed on 2-dimensional imaging, especially pertaining to the temporal bone. Panoramic radiographs have an acceptable specificity for bony change, but poor sensitivity. Thus, if bony disease is suspected and PANO is negative, CBCT may be indicated for supplemental evaluation [96].

CBCT provides an accurate 3-dimensional analysis of the bony components of the temporomandibular joint, free of superimposition or distortion [98]. The benefits of CBCT when compared to CT are that CBCT offers a 3-dimensional analysis in less time, at a lower cost, and with less radiation [99]. In a systematic review, Caruso examined articles advocating the use of CBCT for bony evaluation of the TMJ. The review highlighted the advantages of CBCT, including the ability to calculate condylar volume and surface area. CBCT also offers a comparison between the contralateral condyle for analysis of asymmetry [100]. Linear length, width, and height dimensions of the condyle can be well defined with CBCT, but higher accuracy requires a lower density setting than that recommended for osseous examination. In comparison to lateral cephalograms, posteroanterior, and submentovertex films, CBCT measurements were most accurate and highly reliable, including adequate estimation of the position of the condyle within the glenoid fossa [101].

Hussain conducted a systematic review analyzing panoramic imaging, slice corrected tomography, MRI, CT, high-resolution ultrasound, and CBCT, with osteophyte formation and sclerosis representing body changes. While axially corrected sagittal tomography proved to be the imaging modality of choice, CT did not provide any additionally significant information not otherwise visualized by other modalities. Furthermore, they concluded that combining different radiographic techniques is likely to yield a more accurate diagnosis, and CBCT might prove to be a cost and radiation dose-effective alternative to axially corrected sagittal tomography [97].

### 6.2. Limitations

CBCT is unable to evaluate inflammatory changes of the TMJ, especially in the acute setting [97,102]. Additionally, bone density estimation is poor due to Hounsfield unit distortion. Accordingly, CBCT should only be used when assessing the cortical surface area and dimensions of the condyle and temporal bone. Decreased image quality results in a poor depiction of soft tissue, a well-documentation inadequacy of CBCT. Lastly, deep structural changes of the condylar head, such as pathologic conditions, cysts, and tumors, are poorly visualized with CBCT [70,103].

Despite evidence to suggest certain important applications, there remains a need for definitive, evidence-based data to establish CBCT as the standard imaging modality for the evaluation of the TMJ bony components and disorders. The systematic review conducted by Caruso concluded that although CBCT helped to clarify some aspects of the morphology of the bony aspects of the TMJ, additional evidence-based studies are necessary to assure its validity. In another systematic review, Al-Saleh found very few studies specifying the practical application of CBCT in TMJ disorders, and ultimately could not reach a conclusion regarding accuracy or clinical implications [102].

### 6.3. Conclusion

The advantages of CBCT in the evaluation of the TMJ include an increased diagnostic capability of the bony articular derangements, especially when used to supplement other imaging techniques, such as MRI [93]. Additionally, CBCT offers the clinician the ability to calculate condylar volume and surface area, as well as a comparison between the contralateral condyle [100]. Disadvantages of CBCT were not mentioned in any of the reviewed articles.

When compared to PANO, CBCT offers a higher sensitivity, but specificity is acceptable with PANO [96]. Accuracy was higher in CBCT than lateral cephalograms, PA cephalograms, and PANO. Sensitivity and specificity were not calculated when comparing CBCT to conventional CT. Deviations in treatment and clinical outcome based on the use of CBCT were not concluded in any of the reviewed articles, nor were complication rates. This represents a void in the current literature and certainly an area for future research.

## 7. Orthognathic Surgery

Along with clinical examination, cephalometric radiography has been considered the gold standard for analysis and surgical planning prior to orthognathic surgery [104]. Due to its universal use and acceptance, anatomic landmarks and complex systems of hard and soft tissue analysis have been created based on cephalometric films. Additionally, serial cephalograms allow for growth analysis, which is a critical consideration in timing of surgery.

When conventional cephalometric films have been compared to radiographs constructed from CBCT images, the CBCT radiographs have been found to be accurate and reliable, and of sufficient quality to replace lateral cephalograms [104]. Regardless, in order to rely on CBCT as the sole diagnostic imaging modality, measurement standardization and appropriate landmark identification must be developed in order to mirror or perhaps surpass the positive impact of cephalometric tracings [105].

### 7.1. Preoperative CBCT

#### 7.1.1. Asymmetry

While cephalometric radiographs can accurately depict skeletal discrepancies in the anteroposterior dimension, they are inadequate for the analysis of facial asymmetries, occlusal cants, translational discrepancies, and certain developmental disorders, such as hemifacial microsomia [106,107]. When assessing asymmetries, Jung concluded that 3D evaluation showed a significant advantage compared to lateral cephalograms in hard and soft tissue changes when comparing asymmetric to symmetric cases [106] (Table 3). Lee further demonstrated this discrepancy when analyzing asymmetric patients with 2-dimensional plain films, noting a significant difference in between lateral cephs and CBCT of B points, pogonion, and menton measurements [107].

#### 7.1.2. Soft Tissue

A common critique of CBCT is that it cannot accurately measure soft tissue [92,108]. In regard to orthognathic surgery, soft tissue measurements and predictions are essential in the preoperative analysis and treatment planning phase. In terms of postoperative predictability, calculations remain merely an estimation. Joss et al. conducted a systematic review that declared there to be little to no existing evidence-based literature suggesting any clinical predictability in soft tissue changes [109], (Table 3).

Despite Joss’ conclusions, numerous articles have validated the soft tissue predictability of CBCT. Van Hemelen conducted a double-blinded randomized prospective study to compare the predictive accuracy of 2D planning versus 3D computer aided planning, concluding that the two methods are comparable for both hard and soft tissue profiles [109]. Fourie et al. sought to prove the accuracy of CBCT in measuring soft tissue reference points. In comparing clinical to radiographic measurements, they concluded CBCT provides accurate information regarding soft tissue measurements in the facial region but recommended a 0.3 mm voxel size for added accuracy [92]. In a comparative analysis, Bianchi attempted to validate the predictability of soft tissue esthetics by superimposing pre-operative and post-operative CBCT images [110]. He found an average absolute error of 0.94 mm, with a standard deviation of 0.9 mm, claiming a generalized reliability of CBCT simulations in orthognathic surgery for skeletofacial deformities. Distances between predicted and actual postoperative soft tissue changes were less than 2.0 mm in all assessed regions.

#### 7.1.3. Landmarks

The identification of cephalometric landmarks in 3-dimensional imaging remains variable amongst clinicians and debated in current literature [111]. Lou performed a systematic review to evaluate the available published information on the reliability and accuracy of skeletal landmark identification with computed tomography. For purposes of comparing 3-dimension analysis to 2-dimension analysis in the process of orthognathic surgery planning, eight articles were included. It was determined that each landmark exhibited a characteristic pattern of error that contributed to measurement inaccuracy [112].

Lin investigated the reliability of five horizontal reference planes and numerous landmarks on CBCT, and each of the horizontal reference planes were found to be statistically comparable to conventional imaging [105,112]. Ludlow compared the identification reliability of CBCT and conventional cephalogram tracings, concluding that multi-planar CBCT identifies traditional cephalometric landmarks, such as condylion, gonion, and orbitale more precisely, due to superimposition in 2-dimensional imaging [111]. 3D technique is more favorable than 2D imaging, due to the absence of overlapping anatomical structures and precise measurements of anatomic structures [113].

Notably, other studies have reached opposite conclusions. Van Vlijmen found greater reproducibility of the measurements on conventional cephalometric radiographs compared with the 3D models [114].

While conflicting literature exists, a few studies suggest that CBCT could prove to be more accurate in landmark identification, which could ultimately contribute to increased accuracy in surgical planning [111,113] (Table 3). However, more definitive studies are needed to validate this assertion.

### 7.2. The 3-Dimensional Virtual Surgical Planning and Follow-Up Analysis

Virtual Surgical Planning (VSP) has become prevalent in oral and maxillofacial surgery, as it offers many unique advantages over traditional model surgery. Logistically, planning a surgery virtually prior to anatomic dissection allows for more detailed analysis, avoidance of critical anatomic structures, and ensures confidence in treatment planning [115,116,117]. However, the success and reliability of VSP ultimately relies on the accuracy of the 3-dimensional imaging on which it is based, and the quality of planning software and printing hardware used for fabrication of splints or cutting guides [115,116].

Zhang compared pre-operative CBCT-planned surgery to postoperative clinically measured movements to assess the accuracy of VSP. They found an overall mean linear difference of 0.81 mm (0.71 mm for the maxilla and 0.91 mm for the mandible) and an overall mean angular difference of 0.95 degrees [117]. In a retrospective analysis of over 200 cases, Eggers et al. compared CBCT to CT imaging to assess accuracy in guided surgery. They found the average target registration error was 1.50 ± 0.82 mm with CBCT compared to 1.57 ± 0.84 mm with medical-grade CT image data, indicating statistical equivalence between the two imaging modalities [118].

Most importantly, Gaber et al. conducted a systematic review to definitively assess the validity of 3-dimensional VSP but found no conclusive evidence of its accuracy [115]. Therefore, more conclusive literature is needed indicating the validity of CBCT and its apparent role in surgical planning.

#### 7.2.1. Airway Analysis

CBCT can be used for volumetric analysis of the nasopharynx, oropharynx, and hypopharynx, which can prove beneficial in assessing pre and post-operative treatment for obstructive sleep apnea with or without the use of orthognathic surgery. Sears’ prospective study correlated CBCT to cephalograms before surgery, and 1 month and 6 months after surgery, and determined it to be advantageous to analyze the pharynx at three different levels: the nasopharyx, orophaynx, and velopharynx. Additionally, a weak but statistically significant correlation was found between linear measurements and volumetric analysis [119]. CBCT has also been shown to be accurate in volumetric analysis of the airway, as well as visualization of airway size and shape [119,120,121].

#### 7.2.2. Follow-Up Analysis

Not only is CBCT seemingly useful in the preoperative phase of orthognathic surgery, it can also be used for postoperative 3-dimensional analysis. Compared to 2-dimensional images, such as panoramic and cephalometric plain films, CBCT provides useful information to evaluate success of surgery and measure movements in all dimensions [121].

Wu conducted a comparative study of the outcomes of patients planned with CBCT versus conventional cephalograms in the immediate postoperative phase. Aside from subjective patient satisfaction with CBCT, 3-dimensional planning resulted in superior results in frontal symmetry, change in the angle between the orbital and occlusal lines, frontal ramus inclination, and the distances from the mandibular central incisor and menton to the midsagittal line [122].

Most articles pertaining to post-operative analysis investigate the surgical success as a means of how closely the postoperative results mirror the preoperative plan. However, CBCT importantly also offers 3-dimensional serial analysis over the long term follow up period. Many articles suggest this benefit, but few support this notion with data. [123,124,125].

### 7.3. Conclusion

Advantages of CBCT for its indications in orthognathic surgery include an accurate and reliable analysis, especially when considering facial asymmetry, occlusal cants, translational discrepancies, and certain developmental disorders [106,107]. Soft tissue analysis was proven to be comparable to traditional cephalometric tracing predictions [92,109,110]. Critical landmarks of orthognathic surgery are identified equivalently with CBCT and CT imaging.

Of the numerous articles implementing the use of CBCT in the evaluation of orthognathic surgery, few articles addressed the alteration of treatment and complications. Regarding surgical outcome, CBCT was found to be equivalent to CT for VSP post-surgical comparison.

## 8. Conclusions

The role of CBCT in OMS is variable, due to the significant breadth of the specialty. Ultimately, CBCT provides the depth dimension that cannot be visualized with 2-dimensional films. Although CBCT offers the advantages of a 3-dimensional view with minimal scatter at a low cost and radiation dose when compared with CT and MRI, it is lacking in soft tissue definition and differentiation.

This review confirms that CBCT provides additional information to the clinician in a variety of clinical scenarios. In dentoalveolar surgery, CBCT helps visualize relevant anatomic structures, however limited data exists to suggest a significant change in treatment outcome. Bone volume and linear measurements can be calculated accurately with CBCT, which is useful when preparing for an implant surgery or when assessing osseous lesions. CBCT can also accurately depict bony infections, such as osteomyelitis and osteonecrosis, but falls short to more superior radiographic methods when viewing soft tissue. While some studies advocate the use of CBCT for orthognathic or TMJ surgery, systematic reviews have failed to support their universal application.

CBCT is used for many purposes, including routine OMS procedures, such as removal of impacted teeth and placement of dental implants. However, the quality of literature supporting its use and demonstrating superiority over other imaging methods is quite varied. Although CBCT has now been available for decades, there are many significant opportunities to expand the existing literature and support new applications of this valuable imaging technology.

## Figures and Tables

**Table 1:** Comparison of Cone Beam Computed Tomography to Panoramic Imaging in Dentoalveolar Surgery.

Location	Diagnosis	Alters Treatment	Affects Outcome	Prevents Complications
Mandibular 3rd	Equivalent	Undetermined	Undetermined	Undetermined
Maxillary 3rd	Superior	Undetermined	Undetermined	Undetermined
Impacted Canine	Superior	Yes	Undetermined	Undetermined
Supernumerary	Superior	Undetermined	Undetermined	Undetermined

Undetermined—not enough conclusive evidence or not analyzed.

**Table 2:** Comparison of Cone Beam Computed Tomography to Panoramic Imaging in Dental Implant Surgery.

Implant Criteria	Diagnosis	Change in Treatment	Clinical Outcome	Complications
Anatomy	Superior	Undetermined	Undetermined	Undetermined
Bone Volume/Quality	Superior	Undetermined	Undetermined	Undetermined
Implant	Superior	Variable	Undetermined	Undetermined
Augmentation	Superior	Yes	Undetermined	Undetermined

Variable—conflicting evidence. Undetermined—not enough conclusive evidence or not analyzed.

**Table 3:** Comparison of CBCT to Lateral Cephalograms in Orthognathic Surgery.

Characteristic	Diagnosis	Change in Treatment	Clinical Outcome	Complications
Asymmetry	Superior	Undetermined	Undetermined	Undetermined
Soft Tissue	Equivalent	Undetermined	Undetermined	Undetermined
Landmarks	Variable	Undetermined	Undetermined	Undetermined

Variable—conflicting evidence. Undetermined—not enough conclusive evidence or not analyzed.

## Abbreviations

CBCT	cone beam computed tomography
CT	computed tomography
PANO	panoramic radiography
PA	periapical radiography
MRI	magnetic resonance imaging
TMJ	temporomandibular joint

## References

[1] Ohman A., Kivijarvi K., Blomback U., Flygare L. (2006). Pre-operative radiographic evaluation of lower third molars with computed tomography. Dentomaxillofac. Radiol..

[2] Sekerci A., Sisman Y. (2014). Comparison between panoramic radiography and cone-beam computed tomography findings for assessment of the relationship between impacted mandibular third molars and the mandibular canal. Oral Radiol..

[3] Matzen L.H., Wenzel A. (2015). Efficacy of CBCT for assessment of impacted mandibular third molars: A review Based on a hierarchical model of evidence. Dentomaxillofac. Radiol..

[4] Kosalagood P., Charoenlarp P., Panmekiate S., Sessirisombat S. (2015). Displacement of an impacted maxillary canine root fragment into the nasolacrimal duct: A diagnostic dilemma. J. Oral Maxillofac. Surg. Med. Pathol..

[5] Nakagawa Y., Ishii H., Nomura Y., Watanabe N.Y., Hoshiba D., Kobayashi K., Ishibashi K. (2007). Third Molar Position: Reliability of Panoramic Radiography. J. Oral Maxillofac. Surg..

[6] Rood J., Shehab B.N. (1990). The radiological prediction of inferior alveolar nerve injury during third molar surgery. Br. J. Oral Maxillofac. Surg..

[7] Blaeser B.F., August M.A., Donoff R.B., Kaban L.B., Dodson T.B. (2003). Panoramic radiographic risk factors for inferior alveolar nerve injury after third molar extraction. J. Oral Maxillofac. Surg..

[8] Sedaghatfar M., August M.A., Dodson T.B. (2005). Panoramic radiographic findings as predictors of inferior alveolar nerve exposure following third molar extraction. J. Oral Maxillofac. Surg..

[9] Monaco G., Montevecchi M., Bonetti G.A., Gatto M.R.A., Checchi L., Gatto M.R.A. (2004). Reliability of panoramic radiography in evaluating the topographic relationship between the mandibular canal and impacted third molars. J. Am. Dent. Assoc..

[10] Sun W., Xia K., Tang L., Liu C., Zou L., Liu J. (2018). Accuracy of panoramic radiography in diagnosing maxillary sinus-root relationship: A systematic review and meta-analysis. Angle Orthod..

[11] Ueda M., Nakamori K., Shiratori K., Igarashi T., Sasaki T., Anbo N., Kaneko T., Suzuki N., Dehari H., Sonoda T. (2012). Clinical Significance of Computed Tomographic Assessment and Anatomic Features of the Inferior Alveolar Canal as Risk Factors for Injury of the Inferior Alveolar Nerve at Third Molar Surgery. J. Oral Maxillofac. Surg..

[12] Gomes A.C.A., Vasconcelos B.C.D.E., Silva E.D.D.O., Caldas A.D.F., Neto I.C.P. (2008). Sensitivity and Specificity of Pantomography to Predict Inferior Alveolar Nerve Damage During Extraction of Impacted Lower Third Molars. J. Oral Maxillofac. Surg..

[13] Nakayama K., Nonoyama M., Takaki Y., Kagawa T., Yuasa K., Izumi K., Ozeki S., Ikebe T. (2009). Assessment of the Relationship Between Impacted Mandibular Third Molars and Inferior Alveolar Nerve with Dental 3-Dimensional Computed Tomography. J. Oral Maxillofac. Surg..

[14] Tantanapornkul W., Okochi K., Bhakdinaronk A., Ohbayashi N., Kurabayashi T. (2009). Correlation of darkening of impacted mandibular third molar root on digital panoramic images with cone beam computed tomography findings. Dentomaxillofac. Radiol..

[15] Ghaeminia H., Meijer G.J., Soehardi A., Borstlap W.A., Mulder J., Berge S.J. (2009). Position of the impacted third molar in relation to the mandibular canal. Diagnostic accuracy of cone beam computed tomorgraphy compared with panomaic radiography. Int. J. Oral Maxillofac. Surg..

[16] Guerrero M.E., Botetano R., Beltran J., Horner K., Jacobs R. (2014). Can preoperative imaging help to predict postoperative outcome after wisdom tooth removal? A randomized controlled trial using panoramic radiography versus cone-beam CT. Clin. Oral Investig..

[17] Sharan A., Madjar D. (2006). Correlation between maxillary sinus floor topography and related root position of posterior teeth using panoramic and cross-sectional computed tomography imaging. Oral Surg. Oral Med. Oral Pathol. Oral Radiol. Endodontol..

[18] Jung Y.-H., Cho B.-H. (2012). Assessment of the relationship between the maxillary molars and adjacent structures using cone beam computed tomography. Imaging Sci. Dent..

[19] Eberhardt J.A., Torabinejad M., Christiansen E.L. (1992). A computed tomographic study of the distances between the maxillary sinus floor and the apices of the maxillary posterior teeth. Oral Surg. Oral Med. Oral Pathol..

[20] Bouquet A., Coudert J.-L., Bourgeois D., Mazoyer J.-F., Bossard D. (2004). Contributions of reformatted computed tomography and panoramic radiography in the localization of third molars relative to the maxillary sinus. Oral Surg. Oral Med. Oral Pathol. Oral Radiol. Endodontol..

[21] Hassan B.A. (2010). Reliability of Periapical Radiographs and Orthopantomograms in Detection of Tooth Root Protrusion in the Maxillary Sinus: Correlation Results with Cone Beam Computed Tomography. J. Oral Maxillofac. Res..

[22] Liu D.-G., Zhang W.-L., Zhang Z.-Y., Wu Y.-T., Ma X.-C. (2008). Localization of impacted maxillary canines and observation of adjacent incisor resorption with cone-beam computed tomography. Oral Surg. Oral Med. Oral Pathol. Oral Radiol. Endodontol..

[23] Serrant P.S., McIntyre G.T., Thomson D.J. (2014). Localization of ectopic maxillary canines— Is CBCT more accurate than conventional horizontal or vertical parallax?. J. Orthod..

[24] Preda L., La Fianza A., Di Maggio E., Dore R., Schifino M.R., Campani R., Segù C., Sfondrini M.F., Di Maggio E.M. (1997). The use of spiral computed tomography in the localization of impacted maxillary canines. Dentomaxillofac. Radiol..

[25] Alqerban A., Jacobs R., Fieuws S., Willems G. (2011). Comparison of two cone beam computed tomographic systems versus panoramic imaging for localization of impacted maxillary canines and detection of root resorption. Eur. J. Orthod..

[26] Haney E., Gansky S.A., Lee J.S., Johnson E., Maki K., Miller A.J., Huang J.C. (2010). Comparative analysis of traditional radiographs and cone-beam computed tomography volumetric images in the diagnosis and treatment planning of maxillary impacted canines. Am. J. Orthod. Dentofac. Orthop..

[27] Lai C.S., Bornstein M., Mock L., Heuberger B.M., Dietrich T., Katsaros C. (2013). Impacted maxillary canines and root resorptions of neighbouring teeth: A radiographic analysis using cone-beam computed tomography. Eur. J. Orthod..

[28] Walker L., Enciso R., Mah J. (2005). Three-dimensional localization of maxillary canines with cone-beam computed tomography. Am. J. Orthod. Dentofac. Orthop..

[29] Liu D.-G., Zhang W.-L., Zhang Z.-Y., Wu Y.-T., Ma X.-C. (2007). Three-dimensional evaluations of supernumerary teeth using cone-beam computed tomography for 487 cases. Oral Surg. Oral Med. Oral Pathol. Oral Radiol. Endodontol..

[30] Tyndall D., Brooks S. (2000). Selection criteria for dental implant site imaging: A position paper of the American Academy of Oral and Maxillofacial Radiology. Oral Surg. Oral Med. Oral Pathol. Oral Radiol. Endodontol..

[31] Bokkasam V., Muddepalli P., Jayam R., Devaki S., Pakerla A., Koduri S. (2015). Comparison of panoramic radiograph with cone-beam computed tomography in assessment of maxillary sinus floor and nasal floor. J. Indian Acad. Oral Med. Radiol..

[32] Nowzari H., Molayem S., Chiu C.H.K., Rich S.K. (2010). Cone Beam Computed Tomographic Measurement of Maxillary Central Incisors to Determine Prevalence of Facial Alveolar Bone Width ≥2 mm. Clin. Implant. Dent. Relat. Res..

[33] Yoshimine S.-I., Nishihara K., Nozoe E., Yoshimine M., Nakamura N. (2012). Topographic Analysis of Maxillary Premolars and Molars and Maxillary Sinus Using Cone Beam Computed Tomography. Implant. Dent..

[34] Angelopoulos C., Thomas S., Hechler S., Parissis N., Hlavacek M. (2008). Comparison Between Digital Panoramic Radiography and Cone-Beam Computed Tomography for the Identification of the Mandibular Canal as Part of Presurgical Dental Implant Assessment. J. Oral Maxillofac. Surg..

[35] Uchida Y., Yamashita Y., Goto M., Hanihara T. (2007). Measurement of Anterior Loop Length for the Mandibular Canal and Diameter of the Mandibular Incisive Canal to Avoid Nerve Damage When Installing Endosseous Implants in the Interforaminal Region. J. Oral Maxillofac. Surg..

[36] Gröndahl K., Ekestubbe A., Lofthag-Hansen S., Lofthag-Hansen S. (2009). Cone-Beam CT for Preoperative Implant Planning in the Posterior Mandible: Visibility of Anatomic Landmarks. Clin. Implant Dent. Relat. Res..

[37] Chan H., Brooks S.L., Fu J., Yeh C., Rudek I., Wang H. (2011). Cross-sectional analysis of the mandibular lingual concavity using cone beam computed tomography. Clin. Oral Implant. Res..

[38] Bolin A., Eliasson S., Von Beetzen M., Jansson L. (1996). Radiographic evaluation of mandibular posterior implant sites: Correlation between panoramic and tomographic determinations. Clin. Oral Implant. Res..

[39] Serhal C.B., Persoons M., Hermans R., Van Steenberghe D., Jacobs R., Steenberghe D. (2000). The accuracy of spiral tomography to assess bone quantity for the preoperative planning of implants in the posterior maxilla. Clin. Oral Implant. Res..

[40] Leung C.C., Palomo L., Griffith R., Hans M.G. (2010). Accuracy and reliability of cone-beam computed tomography for measuring alveolar bone height and detecting bony dehiscences and fenestrations. Am. J. Orthod. Dentofac. Orthop..

[41] Kobayashi K., Shimoda S., Nakagawa Y., Yamamoto A. (2004). Accuracy in measurement of distance using limited cone-beam computerized tomography. Int. J. Oral Maxillofac. Implant..

[42] LaScala C., Panella J., Marques M. (2004). Analysis of the accuracy of linear measurements obtained by cone beam computed tomography (CBCT-NewTom). Dentomaxillofac. Radiol..

[43] Mischkowski R.A., Pulsfort R., Ritter L., Neugebauer J., Brochhagen H.G., Keeve E., Zöller J.E. (2007). Geometric accuracy of a newly developed cone-beam device for maxillofacial imaging. Oral Surg. Oral Med. Oral Pathol. Oral Radiol. Endodontol..

[44] Pedroso L., Garcia R.R., Leles J.L., Leles C.R., Silva M.A. (2013). Impact of cone-beam computed tomography on implant planning and on prediction of implant size. Braz. Oral Res..

[45] Schropp L., Stavropoulos A., Gotfredsen E., Wenzel A. (2011). Comparison of panoramic and conventional cross-sectional tomography for preoperative selection of implant size. Clin. Oral Implant. Res..

[46] Correa L.R., Spin-Neto R., Stavropoulos A., Schropp L., Silveira H.E.D., Wenzel A. (2014). Planning of dental implant size with digital panoramic radiographs, CBCT-generated panoramic images and CBCT cross-sectional images. Clin. Oral Implant. Res..

[47] Vazquez L., Saulacic N., Belser U., Bernard J.P. (2008). Efficacy of panoramic radiographs in the preoperative planning of posterior mandibular implants: A prospective clinical study of 1527 consecutively treated patients. Clin. Oral Implant. Res..

[48] Guerrero M.E., Noriega J., Jacobs R. (2014). Preoperative implant planning considering alveolar bone grafting needs and complication prediction using panoramic versus CBCT images. Sci. Dent..

[49] Alkan B.A., Aral C.A., Aral K., Acer N., Şişman Y. (2016). Quantification of circumferential bone level and extraction socket dimensions using different imaging and estimation methods: A comparative study. Oral Radiol..

[50] Baciut M., Hedesiu M., Bran S., Jacobs R., Nackaerts O., Baciut G. (2013). Pre- and postoperative assessment of sinus grafting procedures using cone-beam computed tomography compared with panoramic radiographs. Clin. Oral Implant. Res..

[51] Dorothea C., Dagassan-Berndt N.U., Zitzmann C., Schulze R.K.W. (2015). Implant treatment planning regarding augmentation procedures: Panoramic radiographs vs. cone beam computed tomography images. Clin. Oral Implant. Res..

[52] Neugebauer J., Ritter L., Mischkowski R.A., Dreiseidler T., Scherer P., Ketterle M., Rothamel D., Zöller J.E. (2010). Evaluation of maxillary sinus anatomy by cone-beam CT prior to sinus floor elevation. Int. J. Oral Maxillofac. Implant..

[53] Temmerman A., Hertelé S., Teughels W., Dekeyser C., Jacobs R., Quirynen M. (2011). Are panoramic images reliable in planning sinus augmentation procedures?. Clin. Oral Implant. Res..

[54] Van Steenberghe D., Malevez C., Van Cleynenbreugel J., Bou Serhal C., Dhoore E., Schutyser F., Suetens P., Jacobs R. (2003). Accuracy of drilling guides for transfer from three-dimensional CT-based planning to placement of zygoma implants in human cadavers. Clin. Oral Implant. Res..

[55] Aparicio C. (2011). A proposed classification for zygomatic implant patient based on the zygoma anatomy guided approach (ZAGA): A cross-sectional survey. Eur. J. Oral Implantol..

[56] Fortin T., Champleboux G., Lormée J., Coudert J.L. (2000). Precise dental implant placement in bone using surgical guides in conjunction with medical imaging techniques. J. Oral Implant..

[57] Ganz S.D. (2005). Presurgical Planning with CT-Derived Fabrication of Surgical Guides. J. Oral Maxillofac. Surg..

[58] Jacobs R., Adriansens A., Verstreken K., Suetens P., Van Steenberghe D. (1999). Predictability of a three-dimensional planning system for oral implant surgery. Dentomaxillofac. Radiol..

[59] Nickenig H.-J., Eitner S. (2007). Reliability of implant placement after virtual planning of implant positions using cone beam CT data and surgical (guide) templates. J. Cranio-Maxillofac. Surg..

[60] Ma B., Park T., Chun I., Yun K. (2018). The accuracy of a 3D printing surgical guide determined by CBCT and model analysis. J. Adv. Prosthodont..

[61] E Besimo C., Lambrecht J.T., Guindy J.S. (2000). Accuracy of implant treatment planning utilizing template-guided reformatted computed tomography. Dentomaxillofac. Radiol..

[62] Gallardo Y.N.R., Silva-Olivio I.R.T., Mukai E., Morimoto S., Sesma N., Cordaro L. (2016). Accuracy comparison of guided surgery for dental implants according to the tissue of support: A systematic review and meta-analysis. Clin. Oral Implant. Res..

[63] Schneider D., Marquardt P., Zwahlen M., Jung R.E. (2009). A systematic review on the accuracy and the clinical outcome of computer-guided template-based implant dentistry. Clin. Oral Implant. Res..

[64] Skjerven H., Riis H., Herlofsson B., Ellingsen J. (2019). In Vivo Accuracy of Implant Placement Using a Full Digital Planning Modality and Stereolithographic Guides. Int. J. Oral Maxillofac. Implant..

[65] Nickenig H.J., Wichmann M., Hamel J., Schlegel K.A., Eitner S. (2010). Evaluation of the difference in accuracy between implant placement by virtual planning data and surgical guide templates versus the conventional free-hand method—A combined in vivo—In vitro technique using cone-beam CT (Part II). J. Craniomaxillofac. Surg..

[66] Corpas L.S., Jacobs R., Quirynen M., Huang Y., Naert I., Duyck J. (2011). Peri-implant bone tissue assessment by comparing the outcome of intra-oral radiograph and cone beam computed tomography analyses to the histological standard. Clin. Oral Implant. Res..

[67] Pelekos G., Acharya A., Tonetti M.S., Bornstein M.M. (2018). Diagnostic performance of cone beam computed tomography in assessing peri-implant bone loss: A systematic review. Clin. Oral Implant. Res..

[68] Bohner L.O.L., Mukai E., Oderich E., Porporatti A.L., Pacheco-Pereira C., Tortamano P., Canto G.D.L. (2017). Comparative analysis of imaging techniques for diagnostic accuracy of peri-implant bone defects: A meta-analysis. Oral Surg. Oral Med. Oral Pathol. Oral Radiol..

[69] Jacobs R., Vranckx M., Vanderstuyft T., Quirynen M., Salmon B. (2018). CBCT vs. other imaging modalities to assess peri-implant bone and diagnose complications: A systematic review. Eur. J. Oral Implantol..

[70] Quereshy F.A., Savell T.A., Palomo J.M. (2008). Applications of Cone Beam Computed Tomography in the Practice of Oral and Maxillofacial Surgery. J. Oral Maxillofac. Surg..

[71] Cotti E., Campisi G. (2004). Advanced radiographic techniques for the detection of lesions in bone. Endod. Top..

[72] Mishra S., Degwekar S., Banode P., Bhowate R., Motwani M., Mishra P. (2014). Comparative study of cone-beam computed tomography andmultislice computed tomography in the radiographic evaluation of cysts and tumors of the jaws. J. Indian Acad. Oral Med. Radiol..

[73] Closmann J.J., Schmidt B.L. (2007). The Use of Cone Beam Computed Tomography as an Aid in Evaluating and Treatment Planning for Mandibular Cancer. J. Oral Maxillofac. Surg..

[74] Dreiseidler T., Alarabi N., Ritter L., Rothamel D., Scheer M., Zöller J.E., Mischkowski R.A. (2011). A comparison of multi-slice computerized tomography, cone-beam computerized tomography, and single photon emission computerized tomog-raphy for the assessment of bone invasion by oral malignancies. Oral Surg. Oral Med. Oral Pathol. Oral Radiol. Endod..

[75] Momin M.A., Okochi K., Watanabe H., Imaizumi A., Omura K., Amagasa T., Okada N., Ohbayashi N., Kurabayashi T. (2009). Diagnostic accuracy of cone-beam CT in the assessment of mandibular invasion of lower gingival carcinoma: Comparison with conventional panoramic radiography. Eur. J. Radiol..

[76] Lim L.Z., Padilla R.J., Reside G.J., Tyndall D.A. (2018). Comparing panoramic radiographs and cone beam computed tomography: Impact on radiographic features and differential diagnoses. Oral Surg. Oral Med. Oral Pathol. Oral Radiol..

[77] Chuenchompoonut V., Ida M., Honda E., Kurabayashi T., Sasaki T. (2003). Accuracy of panoramic radiography in assessing the dimensions of radiolucent jaw lesions with distinct or indistinct borders. Dentomaxillofac. Radiol..

[78] Kaneda T., Minami M., Kurabayashi T. (2003). Benign odontogenic tumours of the mandible and maxilla. Neuroimaging Clin. N. Am..

[79] Brauer H., Diaz C., Manegold-Brauer G. (2013). Radiographic assessment of a keratocystic odontogenictumour using cone-beam computed tomography. Eur. Arch. Paediatr. Dent..

[80] Luo J., You M., Zheng G., Xu L. (2014). Cone beam computed tomography signs of desmoplastic ameloblastoma: Review of 7 cases. Oral Surg. Oral Med. Oral Pathol. Oral Radiol..

[81] Araki M., Kameoka S., Mastumoto N., Komiyama K. (2007). Usefulness of cone beam computed tomography for odontogenic myxoma. Dentomaxillofac. Radiol..

[82] Scarfe W.C., Toghyani S., Azevedo B. (2013). Imaging of Benign Odontogenic Lesions. Radiol. Clin. N. Am..

[83] Yoshiura K., Hijiya T., Ariji E., Sa’do B., Nakayama E., Higuchi Y., Kubo S., Ban S., Kanda S. (1994). Radiographic patterns of osteomyelitis in the mandible. Oral Surg. Oral Med. Oral Pathol..

[84] Arce K., Assael L.A., Weissman J.L., Markiewicz M.R. (2009). Imaging findings in bisphosphonate-related osteonecrosis of jaws. J. Oral Maxillofac. Surg..

[85] Olutayo J., Agbaje J.O., Jacobs R., Verhaeghe V., Vande Velde F., Vinckier F. (2010). Bisphosphonate-Related Osteonecrosis of the Jaw Bone: Radiological Pattern and the Potential Role of CBCT in Early Diagnosis. J. Oral Maxillofac. Res..

[86] Treister N.S., Friedland B., Woo S.-B. (2010). Use of cone-beam computerized tomography for evaluation of bisphosphonate-associated osteonecrosis of the jaws. Oral Surg. Oral Med. Oral Pathol. Oral Radiol. Endodontol..

[87] Lofthag-Hansen S., Hummonen S., Gröndahl K., Gröndahl H.-G. (2007). Limited cone-beam CT and intraoral radiography for the diagnosis of periapical pathology. Oral Surg. Oral Med. Oral Pathol. Oral Radiol. Endod..

[88] Fullmer J.M., Scarfe W.C., Kushner G.M., Alpert B., Farman A.G. (2007). Cone beam computed tomographic findings in refractory chronic suppurative osteomyelitis of the mandible. Br. J. Oral Maxillofac. Surg..

[89] Schulze D., Blessmann M., Pohlenz P., Wagner K.W., Heiland M. (2006). Diagnostic criteria for the detection of mandibular osteomyelitis using cone-beam computed tomography. Dentomaxillofac. Radiol..

[90] Yonetsu K., Izumi M., Nakamura T. (1998). Deep facial infections of odontogenic origin: CT assessment of pathways of space involvement. AJNR Am. J. Neuroradiol..

[91] Obayashi N., Ariji Y., Goto M., Izumi M., Naitoh M., Kurita K., Shimozato K., Ariji E. (2004). Spread of odontogenic infection originating in the maxillary teeth: Computerized tomographic assessment. Oral Surg. Oral Med. Oral Pathol. Oral Radiol. Endodontol..

[92] Fourie Z., Damstra J., Gerrits P.O., Ren Y. (2010). Accuracy and reliability of facial soft tissue depth measurements using cone beam computer tomography. Forensic Sci. Int..

[93] Westesson P. (1993). Reliability and validity of imaging diagnosis of temporamandibular joint disorder. Adv. Dent. Res..

[94] Ahmad M., Hollender L., Anderson Q., Kartha K., Ohrbach R.K., Truelove E.L., John M.T., Schiffman E.L. (2009). Research Diagnostic Criteria for Temporomandibular Disorders (RDC/TMD): Development of Image Analysis Criteria and Examiner Reliability for Image Analysis. Oral Surge. Oral Med. Oral Pathol. Oral Radiol. Endodontol..

[95] Al-Saleh M., Punithakumar K., Lagravere M., Boulanger P., Jaremko J.L., Major P.W. (2017). Three-Dimensional Assessment of Temporomandibular Joint Using MRI-CBCT Image Registration. PLoS ONE.

[96] Dahlstrom L., Lindvall A.M. (1996). Assessment of temporomandibular joint disease by panoramic radiography: Reliability and validity in relation to tomography. Dentomaxillofac. Radiol..

[97] Hussain A.M., Packota G., Major P.W., Flores-Mir C. (2008). Role of different imaging modalities in assessment of temporomandibular joint erosions and osteophytes: A systematic review. Dentomaxillofac. Radiol..

[98] Caruso S., Storti E., Nota A., Ehsani S., Gatto R. (2017). Temporomandibular Joint Anatomy Assessed by CBCT Images. BioMed Res. Int..

[99] Tsiklakis K., Syriopoulos K., Stamatakis H. (2004). Radiographic examination of the temporomandibular joint using cone beam computed tomography. Dentomaxillofac. Radiol..

[100] Zhang Y.-L., Song J.-L., Xu X.-C., Zheng L.-L., Wang Q.-Y., Fan Y.-B., Liu Z., Buffoli B. (2016). Morphologic Analysis of the Temporomandibular Joint Between Patients with Facial Asymmetry and Asymptomatic Subjects by 2D and 3D Evaluation. Medicine.

[101] Hilgers M.L., Scarfe W.C., Scheetz J.P., Farman A.G. (2005). Accuracy of linear temporomandibular joint measurements with cone beam computed tomography and digital cephalometric radiography. Am. J. Orthod. Dentofac. Orthop..

[102] Al-Saleh M.A.Q., Alsufyani N.A., Saltaji H., Jaremko J.L., Major P.W. (2016). MRI and CBCT image registration of temporomandibular joint: A systematic review. J. Otolaryngol. Head Neck Surg..

[103] Ahmad M., Jenny J., Downie M. (2012). Application of cone beam computed tomography in oral and maxillofacial surgery. Aust. Dent. J..

[104] Cattaneo P.M., Bloch C.B., Calmar D., Hjortshøj M., Melsen B. (2008). Comparison between conventional and cone-beam computed tomography–generated cephalograms. Am. J. Orthod. Dentofac. Orthop..

[105] Lin H.H., Chuang Y.F., Weng J.L., Lo L.J. (2015). Comparative validity and reproducibility study of various landmark-oriented reference planes in 3-dimensional computed tomogramphic analysis for patients. PLoS ONE.

[106] Jung Y.-J., Kim M.-J., Baek S.-H. (2009). Hard and soft tissue changes after correction of mandibular prognathism and facial asymmetry by mandibular setback surgery: Three-dimensional analysis using computerized tomography. Oral Surg. Oral Med. Oral Pathol. Oral Radiol. Endodontol..

[107] Lee T.-Y., Kim K.-H., Yu H.-S., Kim K.-D., Jung Y.-S., Baik H.-S. (2014). Correlation Analysis of Three-Dimensional Changes of Hard and Soft Tissues in Class III Orthognathic Surgery Patients Using Cone-Beam Computed Tomography. J. Craniofac. Surg..

[108] Van Hemelen G., Van Genechten M., Renier L., Desmedt M., Verbruggen E., Nadjmi N. (2015). Three-dimensional virtual planning in orthognathic surgery enhances the accuracy of soft tissue prediction. J. Cranio-Maxillofac. Surg..

[109] Joss C.U., Joss-Vassalli I.M., Kiliaridis S., Kuijpers-Jagtman A.M. (2010). Soft Tissue Profile Changes After Bilateral Sagittal Split Osteotomy for Mandibular Advancement: A Systematic Review. J. Oral Maxillofac. Surg..

[110] Bianchi A., Muyldermans L., Di Martino M., Lancellotti L., Amadori S., Sarti A., Marchetti C. (2010). Facial Soft Tissue Esthetic Predictions: Validation in Craniomaxillofacial Surgery with Cone Beam Computed Tomography Data. J. Oral Maxillofac. Surg..

[111] Ludlow J.B., Gubler M., Cevidanes L., Mol A. (2009). Precision of cephalometric landmark identification: Cone-beam computed tomography vs. conventional cephalometric views. Am. J. Orthod. Dentofac. Orthop..

[112] Lou L., Lagravère M.O., Compton S., Major P.W., Flores-Mir C. (2007). Accuracy of measurements and reliability of landmark identification with computed tomography (CT) techniques in the maxillofacial area: A systematic review. Oral Surg. Oral Med. Oral Pathol. Oral Radiol. Endodontol..

[113] Farronato G., Salvadori S., Nolet F., Zoia A., Farronato D. (2014). Assessment of inter- and intra-operator cephalometric tracings on cone beam CT radiographs: Comparison of the precision of the cone beam CT versus the latero-lateral radiograph tracing. Prog. Orthod..

[114] Van Vlijmen O.J.C., Maal T., Bergé S.J., Bronkhorst E.M., Katsaros C., Kuijpers-Jagtman A.M. (2010). A comparison between 2D and 3D cephalometry on CBCT scans of human skulls. Int. J. Oral Maxillofac. Surg..

[115] Gaber R.M., Shaheen E., Falter B., Araya S., Politis C., Swennen G.R., Jacobs R. (2017). A systematic review to uncover a universal protocol for accuracy assessment of 3-Dimensional virtually planned orthognathic surgery. J. Oral Maxillofac. Surg..

[116] Xia J., Samman N., Yeung R.W., Shen S.G., Wang D., Ip H.H., Tideman H. (2000). Three-dimensional virtual reality surgical planning and simulation workbench for orthognathic surgery. Int. J. Adult Orthod. Orthognath. Surg..

[117] Zhang N., Liu S., Hu Z., Hu J., Zhu S., Li Y. (2016). Accuracy of virtual surgical planning in two-jaw orthognathic surgery: Comparison of planned and actual results. Oral Surg. Oral Med. Oral Pathol. Oral Radiol..

[118] Eggers G., Senoo H., Kane G., Mühling J. (2009). The accuracy of image guided surgery based on cone beam computer tomography image data. Oral Surg. Oral Med. Oral Pathol. Oral Radiol. Endodontol..

[119] Sears C.R., Miller A.J., Chang M.K., Huang J.C., Lee J.S. (2011). Comparison of Pharyngeal Airway Changes on Plain Radiography and Cone-Beam Computed Tomography after Orthognathic Surgery. J. Oral Maxillofac. Surg..

[120] Abramson Z.R., Susarla S., Tagoni J.R., Kaban L. (2010). Three-Dimensional Computed Tomographic Analysis of Airway Anatomy. J. Oral Maxillofac. Surg..

[121] Ghoneima A., Kula K. (2013). Accuracy and reliability of cone-beam computed tomography for airway volume analysis. Eur. J. Orthod..

[122] Wu T.-Y., Lin H.-H., Lo L.-J., Ho C.-T. (2017). Postoperative outcomes of two- and three-dimensional planning in orthognathic surgery: A comparative study. J. Plast. Reconstr. Aesthet. Surg..

[123] Shaheen E., Shujaat S., Saeed T., Jacobs R., Politis C. (2019). Three-dimensional planning accuracy and follow-up protocol inorthognathic surgery: A validation study. Int. J. Oral Maxillofac. Surg..

[124] De Paula L.K., Ruellas A., Paniagua B., Styner M., Turvey T., Zhu H., Wang J., Cevidanes L., Ruellas A.D.O. (2013). One-year assessment of surgical outcomes in Class III patients using cone beam computed tomography. Int. J. Oral Maxillofac. Surg..

[125] Kim B.-R., Oh K.-M., Cevidanes L.H., Park J.-E., Sim H.-S., Seo S.-K., Reyes M., Kim Y.-J., Park Y.-H. (2013). Analysis of 3D Soft Tissue Changes After 1- and 2-Jaw Orthognathic Surgery in Mandibular Prognathism Patients. J. Oral Maxillofac. Surg..

